# Genes Encoding Microbial Acyl Coenzyme A Binding Protein/Diazepam-Binding Inhibitor Orthologs Are Rare in the Human Gut Microbiome and Show No Links to Obesity

**DOI:** 10.1128/AEM.00471-21

**Published:** 2021-05-26

**Authors:** Andrew Maltez Thomas, Francesco Asnicar, Guido Kroemer, Nicola Segata

**Affiliations:** a Department CIBIO, University of Trento, Trento, Italy; b Université de Paris, Paris, France; c Equipe labellisée par la Ligue contre le Cancer, Centre de Recherche des Cordeliers, Paris, France; d Metabolomics and Cell Biology Platforms, Institut Gustave Roussy, Villejuif, France; e Suzhou Institute for Systems Medicine, Chinese Academy of Medical Sciences, Suzhou, China; f Pôle de Biologie, Hôpital Européen Georges Pompidou, AP-HP, Paris, France; g Karolinska Institute, Department of Women’s and Children’s Health, Karolinska University Hospital, Stockholm, Sweden; h IEO, European Institute of Oncology IRCCS, Milan, Italy; Norwegian University of Life Sciences

**Keywords:** ACBP/DBI, human microbiome

## Abstract

Acyl coenzyme A (CoA) binding protein (ACBP), also called diazepam-binding inhibitor (DBI), is a phylogenetically conserved protein that is expressed by all eukaryotic species as well as by some bacteria. Since elevated ACBP/DBI levels play a major role in the inhibition of autophagy, increase in appetite, and enhanced lipid storage that accompany obesity, we wondered whether ACBP/DBI produced by the human microbiome might affect host weight. We found that the genomes of bacterial commensals rarely contain ACBP/DBI homologues, which are rather encoded by genomes of some pathogenic or environmental taxa that were not prevalent in human feces. Exhaustive bioinformatic analyses of 1,899 gut samples from healthy individuals refuted the hypothesis that bacterial ACBP/DBI might affect the body mass index (BMI) in a physiological context. Thus, the physiological regulation of BMI is unlikely to be affected by microbial ACBP/DBI-like proteins. However, at the speculative level, it remains possible that ACBP/DBI produced by potential pathogenic bacteria might enhance their virulence by inhibiting autophagy and hence subverting innate immune responses.

**IMPORTANCE** Acyl coenzyme A (CoA) binding protein (ACBP) can be encoded by several organisms across the domains of life, including microbes, and has shown to play major roles in human metabolic processes. However, little is known about its presence in the human gut microbiome and whether its microbial counterpart could also play a role in human metabolism. In the present study, we found that microbial ACBP/DBI sequences were rarely present in the gut microbiome across multiple metagenomic data sets. Microbes that carried ACBP/DBI in the human gut microbiome included Saccharomyces cerevisiae, Lautropia mirabilis, and Comamonas kerstersii, but these microorganisms were not associated with body mass index, further indicating an unconvincing role for microbial ACBP/DBI in human metabolism.

## INTRODUCTION

Acyl coenzyme A (CoA) binding protein (ACBP) is also called diazepam-binding inhibitor (DBI). In humans and mice, this small (10 kDa) protein plays a dual role, reflecting its double name. As an intracellular protein, ACBP/DBI binds to medium- and long-chain acyl-CoA esters, reducing their toxicity and facilitating their transport through different subcellular compartments, hence stimulating lipid metabolism (1–3). As an extracellular protein, ACBP/DBI binds to the peripheral benzodiazepine receptor (hence displacing the benzodiazepine diazepam from its binding site), which is the ionotropic gamma-aminobutyric acid type A (GABA_A_) receptor (GABAAR) possessing another endogenous ligand, γ-aminobutyric acid, the major inhibitory neurotransmitter (4, 5). In the central nervous system, ACBP/DBI can be proteolytically cleaved to yield several neuropeptides, one of which, octadecaneuropeptide (ODN), interacts with a G protein coupled receptor (GPCR) in the central nervous system (6, 7).

ACBP/DBI is ubiquitously expressed and can be released from cells through an unconventional, autophagy-dependent pathway (8). It then acts as a paracrine mediator to inhibit autophagy through an action on GABAAR, which is expressed in many cell types outside the central nervous system (9). Hence, antibody-mediated neutralization of extracellular ACBP/DBI offers the possibility to stimulate autophagy by interrupting a paracrine feedback inhibition loop. In humans, obesity and metabolic syndrome are associated with elevated ACBP/DBI levels in the plasma (10), while anorexia nervosa is characterized by abnormally low concentrations of circulating ACBP/DBI (9, 11). In mice, injection of recombinant ACBP/DBI protein into the peritoneal cavity or the tail vein causes a GABAAR-dependent increase in feeding. This appetite-stimulatory effect of ACBP/DBI is also observed for proteins in which the acyl-CoA binding moiety has been mutated. Conversely, injection of a neutralizing antibody blocks feeding responses and counteracts weight gain or favors weight loss in multiple experimental conditions. These findings suggest that ACBP/DBI is involved in the pathophysiology of human obesity (12).

ACBP/DBI is a phylogenetically conserved protein, as ACBP/DBI homologs have been described in all eukaryotic phyla and even in some bacterial species (13, 14). In the nematode Caenorhabditis elegans and in the insect Drosophila melanogaster, ACBP/DBI orthologs stimulate pharyngeal pumping and mouth hook movement, which are the functional equivalents of mammalian mastication (15). In the yeast Saccharomyces cerevisiae, ACBP/DBI is the only protein known to be released in response to nutrient or oxidative stress (16, 17). Extracellular ACBP/DBI stimulates sporulation of yeast in a GPCR-dependent fashion, hence allowing yeast cells to swarm out to find new food resources (15, 18). Thus, the appetite-stimulatory function of ACBP/DBI appears to be phylogenetically conserved (19–21).

Reportedly, the genomes of some bacteria code for ACBP/DBI orthologs (22, 23). It is well known that human obesity is associated with major shifts in the composition of the intestinal microbiome (24, 25). Moreover, fecal microbial transplantation (FMT) of the stools from obese (but not lean) individuals into mice can transfer features of obesity and metabolic syndrome, establishing cause-effect relationships between alterations in the gut microbiome and the obese phenotype (26, 27).

Intrigued by these observations, we wondered whether specific microbial species in the human gut might encode and express ACBP/DBI-like proteins, thus potentially influencing human metabolism and eating behavior. Here, we report a detailed bioinformatics analysis of ACBP/DBI-like genes within the human gut microbiome and analyze their possible implication in obesity. We found that ACBP/DBI is mostly encoded by eukaryotes, that its presence in bacteria is mostly limited to pathogenic taxa, and that its rare presence in the human gut is not associated with alterations in the body mass index (BMI).

## RESULTS

### ACBP/DBI-like proteins are rarely encoded in members of the human microbiome.

To assess whether microbial ACBP/DBI ortholog genes could potentially contribute to microbiome-dependent gut metabolism, we first looked for their presence in 99,211 microbial genomes from NCBI as of January 2019. Using an initial set of 1,098 UniRef-annotated orthologous ACBP/DBI sequences (see Materials and Methods) to search these genomes, we found ACBP to be present in 3,635 of them, encompassing 1,668 unique TaxIDs, with the majority belonging to *Proteobacteria* (89% of genomes). Species with the largest number of genomes encoding ACBP showed it to be part of the core genome of several known pathogens from the *Burkholderia* genus, as well as those from Saccharomyces cerevisiae and Ralstonia solanacearum (Table 1). While Saccharomyces cerevisiae can be found in the human gut (28, 29), although usually at low abundance, the bacterial taxa in NCBI containing ACBP are at best very rare members of the human microbiome.

**TABLE 1:** Top 10 species with the largest number of ACBP/DBI-encoding genomes based on available reference genomes

Species	No. of genomes with ACBP (% positive from genomes searched)
Burkholderia pseudomallei^*a*^	663 (100%)
Burkholderia ubonensis^*a*^	291 (100%)
Burkholderia cenocepacia^*a*^	242 (99.1%)
Burkholderia multivorans^*a*^	198 (100%)
Saccharomyces cerevisiae^*b*^	109 (93.9%)
Burkholderia cepacia^*a*^	98 (100%)
Ralstonia solanacearum^*c*^	80 (100%)
Burkholderia stagnalis^*a*^	64 (100%)
Burkholderia mallei^*a*^	56 (100%)
Burkholderia vietnamiensis^*a*^	44 (100%)

a Taxonomy: *Bacteria*, *Proteobacteria*, *Betaproteobacteria*, *Burkholderiales*, *Burkholderiaceae*, *Burkholderia*.

b Taxonomy: *Eukaryota*, *Ascomycota*, *Saccharomycetes*, *Saccharomycetales*, *Saccharomycetaceae*, *Saccharomyces*.

c Taxonomy: *Bacteria*, *Proteobacteria*, *Betaproteobacteria*, *Burkholderiales*, *Burkholderiaceae*, *Ralstonia*.

Because genomic sequencing captures only a limited fraction of the human microbiome diversity (30–33), we proceeded by searching homologous sequences of known ACBP genes in metagenome-assembled genomes (MAGs). We screened 154,000 MAGs previously recovered from the human microbiome sampled from almost 10,000 individuals spanning diverse geography and lifestyle (Table S1). We found only 129 out of the 154,000 MAGs (0.08%) to encode ACBP, belonging to 14 species-level genome bins (SGBs). One of these SGBs was classified as *Deinococcus-Thermus* and another as *Chitinophagaceae*, whereas the remaining 12 all belonged to *Proteobacteria*, with the closest known taxa being again *Burkholderia* or taxa linked with sample-processing contamination such as *Ralstonia* or *Acidovorax* (34). This exploration of microbial genomes and MAGs thus highlights a lack of ACBP/DBI ortholog genes in microbes of putative relevance in the human microbiome.

### Phylogenetic modeling of ACBP/DBI is highly taxonomically consistent.

**FIG 1:**
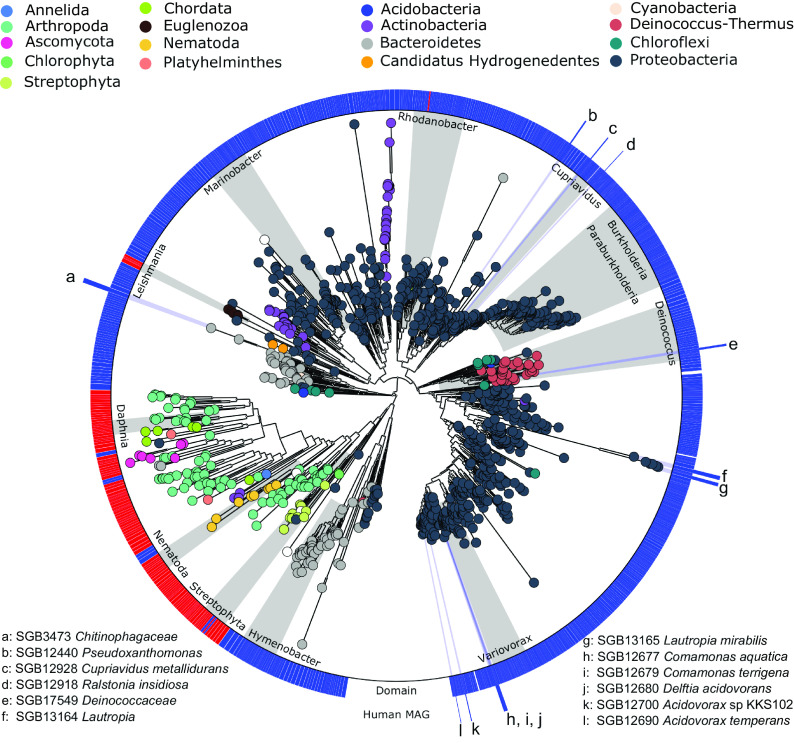
Whole phylogeny of ACBP/DBI gene sequences across kingdoms and phyla. The tree was built using 1,223 ACBP/DBI nucleotide sequences retrieved from UniProtKB, reference genomes from NCBI, and human metagenome-assembled genomes (MAGs) belonging to species-level genome bins (SGBs) from reference 30 (see Materials and Methods). Sequences were clustered at 97% identity prior to multiple sequence alignment and the tree was built using 240 nt of aligned positions.

To better assess the sequence diversity of the ACBP/DBI gene, we phylogenetically modeled its sequence variants found in human MAGs and reference genomes from NCBI across different organisms. This analysis revealed very distinct eukaryotic versus microbial ACBP/DBI sequences, despite the relatively short alignment length used for phylogenetic inference (Fig. 1). This distinct pattern between the two domains was also seen when we used pairwise nucleotide identities calculated from multiple sequence alignments (Fig. S1). We found ACBP to be widespread across the domains of life, with ACBP sequences found in eukaryotic phyla including Streptophyta, Arthropoda, Nematoda, *Ascomycota*, and Chordata and present in 10 different bacterial phyla. Some taxa such as the genera *Daphnia* and *Variovorax* exhibited clearly defined clades, while other taxa such as the phyla Arthropoda and *Actinobacteria* displayed more diverse and paraphyletic phylogenies. The bacterial genera *Burkholderia* and *Paraburkholderia* showed a clearly defined subtree. ACBP sequences belonging to MAGs recovered from the human microbiome were widespread across the phylogeny but always maintained a consistent taxonomic structure. This adherence between phylogeny and taxonomy for ACBP/DBI suggests vertical evolutive trajectories for this gene, as a comparison between prokaryotic phylogenies built at the whole-genome level was highly consistent with the phylogenetic tree constructed for the ACBP/DBI gene (Fig. S2), with very limited evidence (if any) of horizontal transfer events and consequently a low likelihood that yet-to-be-characterized taxa not captured by our analysis carry ACBP/DBI ortholog genes.

### ACBP/DBI is rarely found in human gut microbiomes.

**FIG 2:**
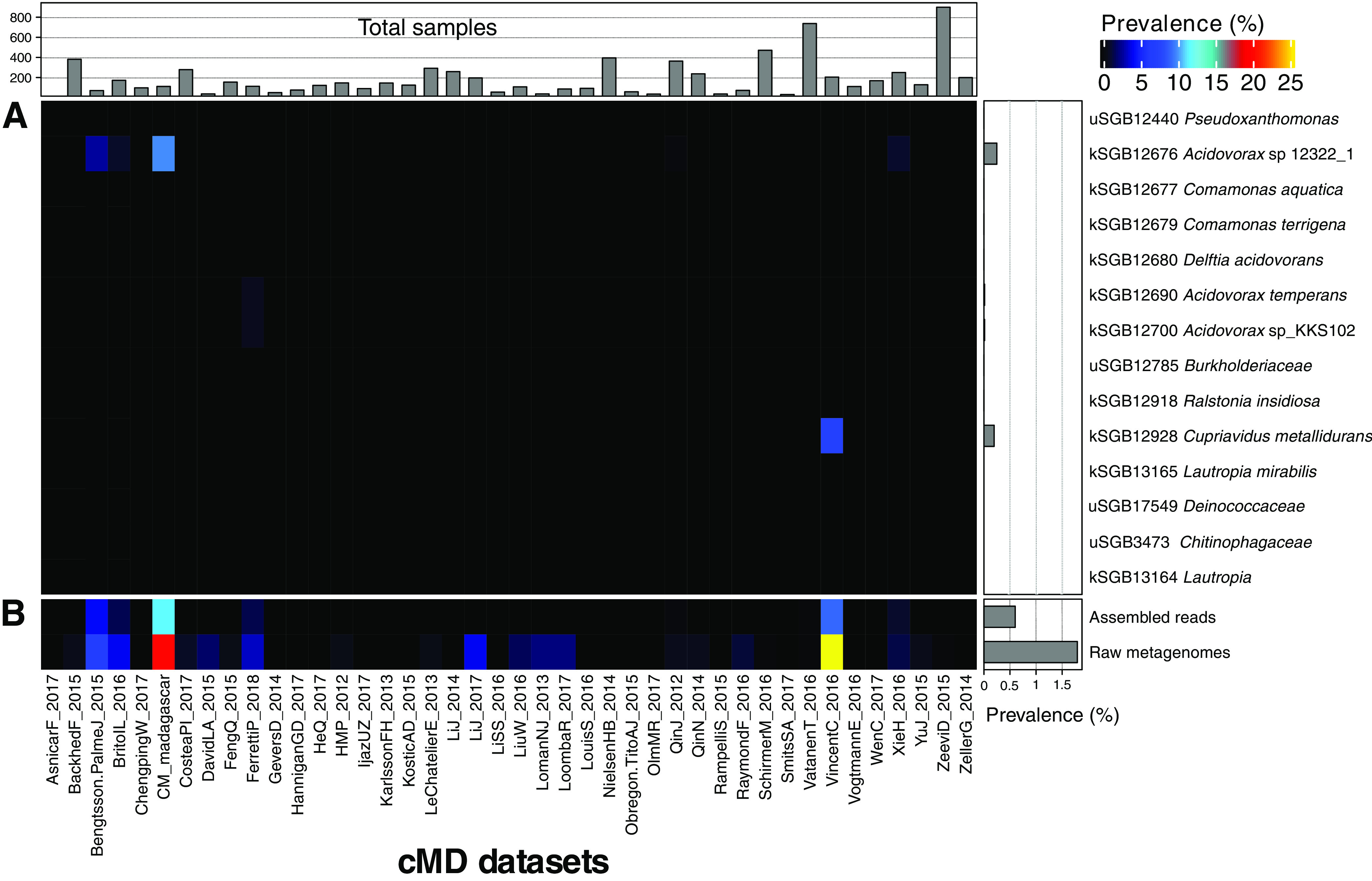
ACBP/DBI is rare in human gut metagenomes. (A) Prevalence of ACBP-encoding SGBs from the human microbiome for all data sets available in curatedMetagenomicData representing 7,698 metagenomic samples from the human gut. (B) The prevalence of assembled reads (contigs) with a significant hit to ACBP sequences and metagenomic reads that map to ACBP sequences with a breadth of coverage of >80% (see Materials and Methods).

To further investigate whether the few ACBP/DBI-positive genomes and MAGs recovered from the human microbiome could potentially contribute to gut metabolism, we evaluated their prevalence across 7,698 human gut metagenomes present in the curatedMetagenomicData R package (35), spanning different countries, age categories, and health conditions (Fig. 2A; Table S2). We found that the majority of MAGs belonging to these SGBs were very rarely found in samples across different data sets, with two known SGBs classified as *Acidovorax* sp. 12322_1 (kSGB 12676) and Cupriavidus metallidurans (kSGB 12928) achieving the highest overall prevalence (0.3%).

Since MAGs rely on the success of metagenomic assembly and binning and thus may miss some low-abundance or hard-to-assemble taxa, we further screened unbinned contigs as well as the raw reads for each sample. The use of unbinned contigs (assembled reads) indeed led to an increase in the overall prevalence of ACBP/DBI-positive samples, but this number remained low (0.6%) (Fig. 2B). When we aligned raw metagenomic reads to the set of retrieved ACBP/DBI sequences, we further observed an increase in the overall relatively low prevalence across samples (1.79%), although we cannot exclude that some of the hits are false positives that inflate the prevalence estimation. Notably, some data sets, such as CM_madagascar from a non-Westernized society (30) and VincentC_2016 comprising fecal microbiome of 98 hospitalized patients treated with antibiotics and that used laxatives (36), showed a higher prevalence of ABCP/DBI in their raw metagenomes compared to others, 19.64% and 25.76%, respectively. On the contrary, 35 data sets (83%) had a prevalence of 0%.

This analysis thus reinforces the very low prevalence of ABCP/DBI-positive taxa and of the ABCP/DBI gene in the human gut microbiome, which appears inconsistent with a hypothesis of a role of this microbial gene variant in human metabolism. Moreover, the taxonomy assignments of the species (from MAGs and NCBI genomes) found to encode ACBP/DBI and occasionally present in some gut microbiome data sets (Fig. 2A) point at sample contamination as a potential source for those taxa. Indeed, *Pseudoxanthomonas*, *Acidovorax*, *Comamonas*, *Delftia*, *Ralstonia*, and *Cupriavidus* have been previously described as common reagent and laboratory contaminants (34).

### Lack of correlation between ACBP/DBI-positive species and body mass index.

**FIG 3:**
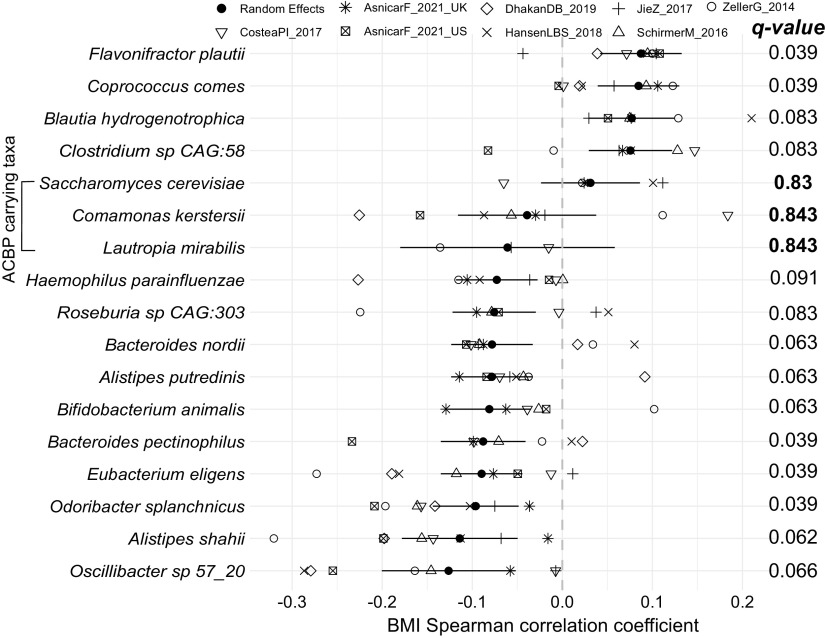
ACBP/DBI-carrying taxa present in the human gut show no significant associations with BMI. We performed a meta-analysis of partial correlations (adjusted for age and sex) between species abundances and BMI across 1,899 samples from healthy gut metagenomes using a random effects model. Meta-analysis *P* values were corrected for multiple hypothesis testing correction using the false discovery rate (*q* values). ACBP-carrying species are shown, as well as species whose FDR is <10%.

Although we found a low prevalence of ACBP/DBI-encoding members in the human gut microbiome, theoretically there could still be a possibility that low-prevalent low-abundance taxa can somehow contribute to human gut metabolism. To evaluate a possible link between microbial ACBP/DBI ortholog genes and obesity, we performed a meta-analysis of correlations between species-level abundances and BMI as a readout using 1,899 gut samples from healthy individuals curated within the curatedMetagenomicData (35) effort (Fig. 3; Table 2). We found 14 taxa to be significantly associated with BMI (random effects model false-discovery rate [FDR] < 0.1) (Table S3), with species such as Flavonifractor plautii, Coprococcus comes, and Blautia hydrogenotrophica associated with increased BMI, in line with previous reports (37, 38). We also found species associated with decreased BMI, which included *Oscillibacter* sp. 57_20, Alistipes shahii, and Odoribacter splanchnicus, as previously described (39). However, these 14 species significantly associated with BMI were all ACBP/DBI-negative. Within the limited panel of ACBP/DBI-positive species at least occasionally found in the gut microbiome, only Saccharomyces cerevisiae, Lautropia mirabilis, and Comamonas kerstersii were sufficiently prevalent in these samples to perform the meta-analysis but showed no significant associations (*q* values > 0.8) (Fig. 3). These results indicate that species found to encode ACBP/DBI in the human gut microbiome do not show associations with BMI.

**TABLE 2:** Demographic information of gut samples from healthy individuals used in the meta-analysis

Data set name	*n*	Age (yrs)			BMI (kg/m^2^)			Sex (*n*)	
		**Mean**	**Min**	**Max**	**Mean**	**Min**	**Max**	**Female**	**Male**
AsnicarF_2021_UK	953	45.6	18.5	65.9	25.3	18.7	40.0	686	267
AsnicarF_2021_US	92	42.5	22.3	65.9	25.9	18.8	38.8	61	31
CosteaPI_2017	82	50.6	29	75	27.4	20.0	38.0	52	30
DhakanDB_2019	80	35.6	19	71	23.6	19.2	36.4	42	38
HansenLBS_2018	57	48.7	22.4	65.4	28.5	21.3	35.1	30	27
JieZ_2017	140	61.0	38	107	23.7	18.8	32.1	76	64
SchirmerM_2016	437	27.8	18	75	22.9	18.8	34.4	246	191
ZellerG_2014	58	61.0	25	84	24.8	20.0	34.0	31	27

## DISCUSSION

ACBP/DBI plays a major role in the control of appetite and metabolism through a phylogenetically conserved pathway that is conserved in yeast, nematodes, insects, and mammals (15, 20, 21, 40). Intrigued by the observation that ACBP/DBI is a highly conserved protein that is even encoded by some bacterial genes, as well as by the link between human obesity and the gut microbiome, we investigated the prevalence of ACBP/DBI in intestinal commensals and their potential correlation with the body mass index.

The bioinformatic analyses presented in this paper based on extensive available metagenomic data sets suggest that ACBP/DBI-producing bacterial species are rather rare in the human microbiome and are mostly produced by eukaryotic species (as exemplified by the yeast S. cerevisiae) and environmental or potentially pathogenic bacteria (exemplified by Comamonas kerstersii that can cause peritonitis, bacteremia, and sepsis [41–43]), as well as potential sample contaminants. Indeed, the presence of ACBP/DBI-producing species in the human gut appears relatively rare. Moreover, we did not find any correlation between the presence of ACBP/DBI-encoding species and BMI across a large cumulative data set comprising 1,899 samples from healthy gut metagenomes. These results refute the hypothesis that the production of ACBP/DBI by the gut microbiome might affect whole-body metabolism, at least in the context of the normal microbiome.

Despite our findings, it could still be possible that microbes that are strongly associated with the mucosal tissue in the upper intestinal tract (and that hence would be grossly underrepresented in fecal samples) might have some local or systemic effects. It is also noteworthy to mention that the lack of an association between ACBP/DBI gene carriage and obesity found here did not take into account gene expression levels, which could be relevant as they might not mirror gene presence and/or abundance patterns. Moreover, in the context of infections, bacterial ACBP/DBI might exert some physiological effects on the host. However, it is unclear whether prokaryotic ACBP/DBI orthologues possess similar functions as those present in yeast or other eukaryotes, despite previous work showing strong conservation of amino acids at the majority of sites determined to be important for ACBP structure and function across phyla (22). ACBP/DBI inhibits autophagy (9, 19), and autophagy is a potent mechanism to eliminate intracellular bacteria (44), meaning that the subversion of autophagy (also called xenophagy) might contribute to the virulence of pathogenic species. Thus, Streptococcus pneumoniae degrades the essential autophagy protein ATG14 to ensure its survival in host cells (45), while Salmonella enterica serovar Typhimurium targets the V-ATPase-ATG16L1 axis to avoid xenophagy (46), just to mention a few examples. In view of these premises, it might be interesting to generate recombinant bacterial ACBP/DBI proteins and to evaluate them for their autophagy-inhibitory and metabolic effects.

The appetite-stimulatory effects of ACBP/DBI are lost in mice that bear a phenylalanine (F) to isoleucine (I) substitution at position 77 in the N-terminal domain of the gamma2 subunit of GABAAR (10, 47), supporting the contention that this neurotransmitter receptor is responsible for the obesogenic activity of DBI. ACBP/DBI is a GABAAR antagonist, while GABA is a GABAAR agonist. Of note, GABA, the natural agonist of GABAAR, can be produced by a series of bacteria. Reportedly, oral administration of GABA-producing Lactobacillus brevis strains reduces the abundance of mesenteric adipose tissue, enhances insulin secretion following glucose challenge, and improves plasma cholesterol clearance (48). Hence, it is possible that, beyond their documented effects on depression (49, 50), GABA-producing bacteria might affect whole-body metabolism, including appetite control. This hypothesis will be actively investigated by our laboratories.

## MATERIALS AND METHODS

### Identification of ACBP/DBI sequences and phylogenetic tree reconstruction.

To obtain a more comprehensive set of ACBP/DBI sequences, we downloaded amino acid sequences that matched the keyword “ACBP” from UniProt90 (51), mapped their identifiers to those of the European Molecular Biology Laboratory’s coding sequences using UniParc, and used the resulting DNA sequences to search, using BLASTn (52), all 99,211 microbial genomes available in NCBI, that included the whole set of 17,607 microbial species (16,959 bacteria, 648 archaea) available as of January 2019 and 154,723 metagenome-assembled genomes (MAGs) from reference 30. Matching queries were filtered to include only alignments with >70% identity, alignment length of >100 nt, and an E value of <1 × 10^−5^. We found no evidence that more permissive minimum alignment lengths lead to increased ACBP/DBI detection.

To build a phylogenetic tree of the known and metagenomically retrieved sequences, we clustered sequences at 97% sequence identity using UCLUST (parameters: “-id 0.97”) (53) and aligned centroid cluster sequences using MAFFT (parameters: “–localpair –maxiterate 1000”) (54). We removed “gappy” regions and ACBP/DBI sequences with insufficient aligned positions from the multiple sequence alignment using Jalview (55), resulting in 240 nucleotides of aligned positions and 1,223 sequences. The tree was built using fastTree (parameters: “-mlacc 2 -slownni -spr 4 -fastest -mlnni 4 -no2nd -nt”) (56) and refined with RAxML (parameters: “-m GTRGAMMA -t”) (57). GraPhlAn (58) was used for tree annotation and visualization.

We used PhyloPhlAn 3 (59) to build a phylogeny on 3,490 reference prokaryotic genomes and 129 MAGs (which we found to contain ACBP/DBI) using the parameters “-diversity high –accurate –force_nucleotides” and the set of up to 400 PhyloPhlAn genome markers. We compared trees built using PhyloPhlAn 3 and ACBP/DBI (with the aforementioned methods) in terms of their normalized pairwise branch lengths and used the tqDist (60) function available in the R quartet package to compare their quartet distances using a random sampling of 477 genomes repeated 1,000 times.

### Search of ACBP/DBI sequences in human gut metagenomes.

The prevalence of both known and unknown species-level genome bins (kSGBs and uSGBs) that were found in a repository (https://opendata.lifebit.ai/table/SGB) (30) with ACBP/DBI-encoding MAGs was calculated using 7,698 human gut metagenomes present in the curatedMetagenomicData (cMD) version 1.16.0 R package (35). A given sample was deemed positive if a MAG belonging to the ACBP/DBI-encoding SGB was found.

We used the set of retrieved ACBP/DBI sequences to search, using BLASTn, all contigs assembled from human gut metagenomes available in cMD. Samples were considered to be positive for ACBP if any of their contigs had a significant hit (>70% identity, alignment length of >100 nt, and an E value of <1 × 10^−5^).

We aligned raw reads from these gut metagenomes to the set of retrieved ACBP/DBI sequences using bowtie2 (61). Resulting BAM files were filtered to keep only alignments with more than 50 nt of matching positions and were used to calculate the breadth of coverage of each sequence using Samtools (62) and VCF utils (63). Samples whose metagenome presented ACBP/DBI sequences with breadth of >80% were considered positive.

### Correlations between BMI and species’ abundances.

We used the PREDICT 1 data set comprising 1,001 healthy individuals from the UK and 97 from the US (38), as well as publicly available data sets collected in cMD and profiled with version 3 of MetaPhlAn (64, 65). Of the 57 data sets available, we selected those that had samples with the following characteristics: (i) gut samples collected from healthy adult individuals at first collection (“days_from_first_collection” = 0 or not available [NA]) and (ii) samples with age, sex, and BMI data available. Outlier samples were removed if their BMI value was outside 3.5 and 7.5 times the interquartile range (IQR) of samples meeting the above criteria (IQR = 5.03). Only data sets with at least 50 samples were considered: Asnicar_2020_UK (953 samples out of 1,001), Asnicar_2020_US (92 samples out of 97) (38), CosteaPI_2017 (82 samples out of 279) (66), DhakanDB_2019 (80 samples out of 110) (67), HansenLBS_2018 (57 samples out of 208) (68), JieZ_2017 (140 samples out of 385) (39), SchirmerM_2016 (437 samples out of 471) (69), and ZellerG_2014 (58 samples out of 199) (70).

For each species, Spearman’s correlations with BMI were computed using the pcor.test function from the ppcor R package controlling for age and sex. Resulting correlations were used as input to the metacor function from the meta R package using Fisher’s Z transformation of correlations and the Paule-Mandel estimator of between-study variance in the random effects model. *P* values from the random-effects model were corrected using false discovery rate (FDR) through the Benjamini-Hochberg procedure, which are reported in the figure as *q* values. We report *q* values of ACBP/DBI-carrying taxa found in these data sets, as well as those of species with FDR of <0.1.

## References

[1] Mandrup S, Højrup P, Kristiansen K, Knudsen J. 1991. Gene synthesis, expression in Escherichia coli, purification and characterization of the recombinant bovine acyl-CoA-binding protein. Biochem J 276:817–823. 10.1042/bj2760817. 2064616 PMC1151077

[2] Duman C, Yaqubi K, Hoffmann A, Acikgöz AA, Korshunov A, Bendszus M, Herold-Mende C, Liu H-K, Alfonso J. 2019. Acyl-CoA-binding protein drives glioblastoma tumorigenesis by sustaining fatty acid oxidation. Cell Metab 30:274–289.e5. 10.1016/j.cmet.2019.04.004. 31056285

[3] Qiu S, Zeng B. 2020. Advances in understanding the acyl-CoA-binding protein in plants, mammals, yeast, and filamentous fungi. J Fungi (Basel) 6:34. 10.3390/jof6010034. PMC7151191 32164164

[4] Knudsen J, Mandrup S, Rasmussen JT, Andreasen PH, Poulsen F, Kristiansen K. 1993. The function of acyl-CoA-binding protein (ACBP)/diazepam binding inhibitor (DBI). Mol Cell Biochem 123:129–138. 10.1007/BF01076484. 8232254

[5] Tonon M-C, Vaudry H, Chuquet J, Guillebaud F, Fan J, Masmoudi-Kouki O, Vaudry D, Lanfray D, Morin F, Prevot V, Papadopoulos V, Troadec J-D, Leprince J. 2020. Endozepines and their receptors: structure, functions and pathophysiological significance. Pharmacol Ther 208:107386. 10.1016/j.pharmthera.2019.06.008. 31283949

[6] de Mateos-Verchere JG, Leprince J, Tonon MC, Vaudry H, Costentin J. 2001. The octadecaneuropeptide [diazepam-binding inhibitor (33–50)] exerts potent anorexigenic effects in rodents. Eur J Pharmacol 414:225–231. 10.1016/S0014-2999(01)00771-3. 11239923

[7] Bouyakdan K, Martin H, Liénard F, Budry L, Taib B, Rodaros D, Chrétien C, Biron É, Husson Z, Cota D, Pénicaud L, Fulton S, Fioramonti X, Alquier T. 2019. The gliotransmitter ACBP controls feeding and energy homeostasis via the melanocortin system. J Clin Invest 129:2417–2430. 10.1172/JCI123454. 30938715 PMC6546475

[8] Loomis WF, Behrens MM, Williams ME, Anjard C. 2010. Pregnenolone sulfate and cortisol induce secretion of acyl-CoA-binding protein and its conversion into endozepines from astrocytes. J Biol Chem 285:21359–21365. 10.1074/jbc.M110.105858. 20452969 PMC2898429

[9] Bravo-San Pedro JM, Sica V, Martins I, Pol J, Loos F, Maiuri MC, Durand S, Bossut N, Aprahamian F, Anagnostopoulos G, Niso-Santano M, Aranda F, Ramírez-Pardo I, Lallement J, Denom J, Boedec E, Gorwood P, Ramoz N, Clément K, Pelloux V, Rohia A, Pattou F, Raverdy V, Caiazzo R, Denis RGP, Boya P, Galluzzi L, Madeo F, Migrenne-Li S, Cruciani-Guglielmacci C, Tavernarakis N, López-Otín C, Magnan C, Kroemer G. 2019. Acyl-CoA-binding protein is a lipogenic factor that triggers food intake and obesity. Cell Metab 30:754–767.e9. 10.1016/j.cmet.2019.07.010. 31422903

[10] Joseph A, Moriceau S, Sica V, Anagnostopoulos G, Pol J, Martins I, Lafarge A, Maiuri MC, Leboyer M, Loftus J, Bellivier F, Belzeaux R, Berna F, Etain B, Capdevielle D, Courtet P, Dubertret C, Dubreucq J, Thierry DA, Fond G, Gard S, Llorca P-M, Mallet J, Misdrahi D, Olié E, Passerieux C, Polosan M, Roux P, Samalin L, Schürhoff F, Schwan R, Magnan C, Oury F, Bravo-San Pedro JM, Kroemer G. 2020. Metabolic and psychiatric effects of acyl coenzyme A binding protein (ACBP)/diazepam binding inhibitor (DBI). Cell Death Dis 11:502. 10.1038/s41419-020-2716-5. 32632162 PMC7338362

[11] Conti E, Tremolizzo L, Bomba M, Uccellini O, Rossi MS, Raggi ME, Neri F, Ferrarese C, Nacinovich R. 2013. Reduced fasting plasma levels of diazepam-binding inhibitor in adolescents with anorexia nervosa. Int J Eat Disord 46:626–629. 10.1002/eat.22129. 23625555

[12] Sica V, Martins I, Motiño O, Bravo-San Pedro JM, Kroemer G. 2020. Antibody-mediated neutralization of ACBP/DBI has anorexigenic and lipolytic effects. Adipocyte 9:116–119. 10.1080/21623945.2020.1736734. 32157940 PMC7153538

[13] Rose TM, Schultz ER, Todaro GJ. 1992. Molecular cloning of the gene for the yeast homolog (ACB) of diazepam binding inhibitor/endozepine/acyl-CoA-binding protein. Proc Natl Acad Sci U S A 89:11287–11291. 10.1073/pnas.89.23.11287. 1454809 PMC50535

[14] Faergeman NJ, Wadum M, Feddersen S, Burton M, Kragelund BB, Knudsen J. 2007. Acyl-CoA binding proteins; structural and functional conservation over 2000 MYA. Mol Cell Biochem 299:55–65. 10.1007/s11010-005-9040-3. 17013545

[15] Charmpilas N, Ruckenstuhl C, Sica V, Büttner S, Habernig L, Dichtinger S, Madeo F, Tavernarakis N, Bravo-San Pedro JM, Kroemer G. 2020. Acyl-CoA-binding protein (ACBP): a phylogenetically conserved appetite stimulator. Cell Death Dis 11:7. 10.1038/s41419-019-2205-x. 31907349 PMC6944704

[16] Cruz-Garcia D, Brouwers N, Malhotra V, Curwin AJ. 2020. Reactive oxygen species triggers unconventional secretion of antioxidants and Acb1. J Cell Biol 219:e201905028. 10.1083/jcb.201905028. 32328640 PMC7147093

[17] Duran JM, Anjard C, Stefan C, Loomis WF, Malhotra V. 2010. Unconventional secretion of Acb1 is mediated by autophagosomes. J Cell Biol 188:527–536. 10.1083/jcb.200911154. 20156967 PMC2828925

[18] Manjithaya R, Anjard C, Loomis WF, Subramani S. 2010. Unconventional secretion of Pichia pastoris Acb1 is dependent on GRASP protein, peroxisomal functions, and autophagosome formation. J Cell Biol 188:537–546. 10.1083/jcb.200911149. 20156962 PMC2828923

[19] Bravo-San Pedro JM, Sica V, Martins I, Anagnostopoulos G, Maiuri C, Kroemer G. 2019. Cell-autonomous, paracrine and neuroendocrine feedback regulation of autophagy by DBI/ACBP (diazepam binding inhibitor, acyl-CoA binding protein): the obesity factor. Autophagy 15:2036–2038. 10.1080/15548627.2019.1662585. 31470770 PMC6844558

[20] Bravo-San Pedro JM, Sica V, Kroemer G. 2019. The elusive “hunger protein”: an appetite-stimulatory factor that is overabundant in human obesity. Mol Cell Oncol 6:e1667193. 10.1080/23723556.2019.1667193. 31692883 PMC6816372

[21] Madeo F, Tavernarakis N, Pedro J-S, Kroemer G. 2020. ACBP is an appetite stimulator across phylogenetic barriers. Cell Stress 4:27–29. 10.15698/cst2020.02.211. 32043075 PMC6997948

[22] Burton M, Rose TM, Faergeman NJ, Knudsen J. 2005. Evolution of the acyl-CoA binding protein (ACBP). Biochem J 392:299–307. 10.1042/BJ20050664. 16018771 PMC1316265

[23] Raboanatahiry NH, Lu G, Li M. 2015. Computational prediction of acyl-coA binding proteins structure in Brassica napus. PLoS One 10:e0129650. 10.1371/journal.pone.0129650. 26065422 PMC4465970

[24] Goodrich JK, Waters JL, Poole AC, Sutter JL, Koren O, Blekhman R, Beaumont M, Van Treuren W, Knight R, Bell JT, Spector TD, Clark AG, Ley RE. 2014. Human genetics shape the gut microbiome. Cell 159:789–799. 10.1016/j.cell.2014.09.053. 25417156 PMC4255478

[25] Sonnenburg JL, Bäckhed F. 2016. Diet-microbiota interactions as moderators of human metabolism. Nature 535:56–64. 10.1038/nature18846. 27383980 PMC5991619

[26] Ridaura VK, Faith JJ, Rey FE, Cheng J, Duncan AE, Kau AL, Griffin NW, Lombard V, Henrissat B, Bain JR, Muehlbauer MJ, Ilkayeva O, Semenkovich CF, Funai K, Hayashi DK, Lyle BJ, Martini MC, Ursell LK, Clemente JC, Van Treuren W, Walters WA, Knight R, Newgard CB, Heath AC, Gordon JI. 2013. Gut microbiota from twins discordant for obesity modulate metabolism in mice. Science 341:1241214. 10.1126/science.1241214. 24009397 PMC3829625

[27] de Groot P, Scheithauer T, Bakker GJ, Prodan A, Levin E, Khan MT, Herrema H, Ackermans M, Serlie MJM, de Brauw M, Levels JHM, Sales A, Gerdes VE, Ståhlman M, Schimmel AWM, Dallinga-Thie G, Bergman JJ, Holleman F, Hoekstra JBL, Groen A, Bäckhed F, Nieuwdorp M. 2020. Donor metabolic characteristics drive effects of faecal microbiota transplantation on recipient insulin sensitivity, energy expenditure and intestinal transit time. Gut 69:502–512. 10.1136/gutjnl-2019-318320. 31147381 PMC7034343

[28] Nash AK, Auchtung TA, Wong MC, Smith DP, Gesell JR, Ross MC, Stewart CJ, Metcalf GA, Muzny DM, Gibbs RA, Ajami NJ, Petrosino JF. 2017. The gut mycobiome of the Human Microbiome Project healthy cohort. Microbiome 5:153. 10.1186/s40168-017-0373-4. 29178920 PMC5702186

[29] Hallen-Adams HE, Suhr MJ. 2017. Fungi in the healthy human gastrointestinal tract. Virulence 8:352–358. 10.1080/21505594.2016.1247140. 27736307 PMC5411236

[30] Pasolli E, Asnicar F, Manara S, Zolfo M, Karcher N, Armanini F, Beghini F, Manghi P, Tett A, Ghensi P, Collado MC, Rice BL, DuLong C, Morgan XC, Golden CD, Quince C, Huttenhower C, Segata N. 2019. Extensive unexplored human microbiome diversity revealed by over 150,000 genomes from metagenomes spanning age, geography, and lifestyle. Cell 176:649–662.e20. 10.1016/j.cell.2019.01.001. 30661755 PMC6349461

[31] Almeida A, Nayfach S, Boland M, Strozzi F, Beracochea M, Shi ZJ, Pollard KS, Sakharova E, Parks DH, Hugenholtz P, Segata N, Kyrpides NC, Finn RD. 2021. A unified catalog of 204,938 reference genomes from the human gut microbiome. Nat Biotechnol 39:105–114. 10.1038/s41587-020-0603-3. 32690973 PMC7801254

[32] Almeida A, Mitchell AL, Boland M, Forster SC, Gloor GB, Tarkowska A, Lawley TD, Finn RD. 2019. A new genomic blueprint of the human gut microbiota. Nature 568:499–504. 10.1038/s41586-019-0965-1. 30745586 PMC6784870

[33] Nayfach S, Shi ZJ, Seshadri R, Pollard KS, Kyrpides NC. 2019. New insights from uncultivated genomes of the global human gut microbiome. Nature 568:505–510. 10.1038/s41586-019-1058-x. 30867587 PMC6784871

[34] Salter SJ, Cox MJ, Turek EM, Calus ST, Cookson WO, Moffatt MF, Turner P, Parkhill J, Loman NJ, Walker AW. 2014. Reagent and laboratory contamination can critically impact sequence-based microbiome analyses. BMC Biol 12:87. 10.1186/s12915-014-0087-z. 25387460 PMC4228153

[35] Pasolli E, Schiffer L, Manghi P, Renson A, Obenchain V, Truong DT, Beghini F, Malik F, Ramos M, Dowd JB, Huttenhower C, Morgan M, Segata N, Waldron L. 2017. Accessible, curated metagenomic data through ExperimentHub. Nat Methods 14:1023–1024. 10.1038/nmeth.4468. 29088129 PMC5862039

[36] Vincent C, Miller MA, Edens TJ, Mehrotra S, Dewar K, Manges AR. 2016. Bloom and bust: intestinal microbiota dynamics in response to hospital exposures and Clostridium difficile colonization or infection. Microbiome 4:12. 10.1186/s40168-016-0156-3. 26975510 PMC4791782

[37] Gomes AC, Hoffmann C, Mota JF. 2018. The human gut microbiota: metabolism and perspective in obesity. Gut Microbes 9:308–325. 10.1080/19490976.2018.1465157. 29667480 PMC6219651

[38] Asnicar F, Berry SE, Valdes AM, Nguyen LH, Piccinno G, Drew DA, Leeming E, Gibson R, Le Roy C, Khatib HA, Francis L, Mazidi M, Mompeo O, Valles-Colomer M, Tett A, Beghini F, Dubois L, Bazzani D, Thomas AM, Mirzayi C, Khleborodova A, Oh S, Hine R, Bonnett C, Capdevila J, Danzanvilliers S, Giordano F, Geistlinger L, Waldron L, Davies R, Hadjigeorgiou G, Wolf J, Ordovás JM, Gardner C, Franks PW, Chan AT, Huttenhower C, Spector TD, Segata N. 2021. Microbiome connections with host metabolism and habitual diet from 1,098 deeply phenotyped individuals. Nat Med 27:321–332. 10.1038/s41591-020-01183-8. 33432175 PMC8353542

[39] Jie Z, Xia H, Zhong S-L, Feng Q, Li S, Liang S, Zhong H, Liu Z, Gao Y, Zhao H, Zhang D, Su Z, Fang Z, Lan Z, Li J, Xiao L, Li J, Li R, Li X, Li F, Ren H, Huang Y, Peng Y, Li G, Wen B, Dong B, Chen J-Y, Geng Q-S, Zhang Z-W, Yang H, Wang J, Wang J, Zhang X, Madsen L, Brix S, Ning G, Xu X, Liu X, Hou Y, Jia H, He K, Kristiansen K. 2017. The gut microbiome in atherosclerotic cardiovascular disease. Nat Commun 8:845. 10.1038/s41467-017-00900-1. 29018189 PMC5635030

[40] Pedro J-S, Sica V, Madeo F, Kroemer G. 2019. Acyl-CoA-binding protein (ACBP): the elusive “hunger factor” linking autophagy to food intake. Cell Stress Chaperones 3:312–318. 10.15698/cst2019.10.200. PMC6789435 31656948

[41] Kaeuffer C, Schramm F, Meyer A, Hansmann Y, Guffroy A, Argemi X. 2018. First case of Comamonas aquatica bacteremia complicated by septic shock. Med Mal Infect 48:540–542. 10.1016/j.medmal.2018.08.004. 30270173

[42] Tiwari S, Nanda M. 2019. Bacteremia caused by Comamonas testosteroni an unusual pathogen. J Lab Physicians 11:87–90. 10.4103/JLP.JLP_116_18. 30983809 PMC6437828

[43] Liu X-J, Qiao X-W, Huang T-M, Li L, Jiang S-P. 2020. Comamonas kerstersii bacteremia. Med Mal Infect 50:288–290. 10.1016/j.medmal.2019.12.005. 32169298

[44] Levine B, Kroemer G. 2019. Biological functions of autophagy genes: a disease perspective. Cell 176:11–42. 10.1016/j.cell.2018.09.048. 30633901 PMC6347410

[45] Shizukuishi S, Ogawa M, Ryo A, Ohnishi M. 2020. Streptococcus pneumoniae promotes its own survival via choline-binding protein CbpC-mediateddegradation of ATG14. Autophagy 16:1529–1531. 10.1080/15548627.2020.1776475. 32508214 PMC7480810

[46] Xu Y, Zhou P, Cheng S, Lu Q, Nowak K, Hopp A-K, Li L, Shi X, Zhou Z, Gao W, Li D, He H, Liu X, Ding J, Hottiger MO, Shao F. 2019. A bacterial effector reveals the V-ATPase-ATG16L1 axis that initiates xenophagy. Cell 178:552–566.e20. 10.1016/j.cell.2019.06.007. 31327526

[47] Dumitru I, Neitz A, Alfonso J, Monyer H. 2017. Diazepam binding inhibitor promotes stem cell expansion controlling environment-dependent neurogenesis. Neuron 94:125–137.e5. 10.1016/j.neuron.2017.03.003. 28343864

[48] Patterson E, Ryan PM, Wiley N, Carafa I, Sherwin E, Moloney G, Franciosi E, Mandal R, Wishart DS, Tuohy K, Ross RP, Cryan JF, Dinan TG, Stanton C. 2019. Gamma-aminobutyric acid-producing lactobacilli positively affect metabolism and depressive-like behaviour in a mouse model of metabolic syndrome. Sci Rep 9:16323. 10.1038/s41598-019-51781-x. 31704943 PMC6841999

[49] Strandwitz P, Kim KH, Terekhova D, Liu JK, Sharma A, Levering J, McDonald D, Dietrich D, Ramadhar TR, Lekbua A, Mroue N, Liston C, Stewart EJ, Dubin MJ, Zengler K, Knight R, Gilbert JA, Clardy J, Lewis K. 2019. GABA-modulating bacteria of the human gut microbiota. Nat Microbiol 4:396–403. 10.1038/s41564-018-0307-3. 30531975 PMC6384127

[50] Valles-Colomer M, Falony G, Darzi Y, Tigchelaar EF, Wang J, Tito RY, Schiweck C, Kurilshikov A, Joossens M, Wijmenga C, Claes S, Van Oudenhove L, Zhernakova A, Vieira-Silva S, Raes J. 2019. The neuroactive potential of the human gut microbiota in quality of life and depression. Nat Microbiol 4:623–632. 10.1038/s41564-018-0337-x. 30718848

[51] Apweiler R, Bairoch A, Wu CH, Barker WC, Boeckmann B, Ferro S, Gasteiger E, Huang H, Lopez R, Magrane M, Martin MJ, Natale DA, O'Donovan C, Redaschi N, Yeh L-SL. 2004. UniProt: the Universal Protein knowledgebase. Nucleic Acids Res 32:D115–D119. 10.1093/nar/gkh131. 14681372 PMC308865

[52] Altschul SF, Gish W, Miller W, Myers EW, Lipman DJ. 1990. Basic local alignment search tool. J Mol Biol 215:403–410. 10.1016/S0022-2836(05)80360-2. 2231712

[53] Edgar RC. 2010. Search and clustering orders of magnitude faster than BLAST. Bioinformatics 26:2460–2461. 10.1093/bioinformatics/btq461. 20709691

[54] Katoh K, Standley DM. 2013. MAFFT multiple sequence alignment software version 7: improvements in performance and usability. Mol Biol Evol 30:772–780. 10.1093/molbev/mst010. 23329690 PMC3603318

[55] Waterhouse AM, Procter JB, Martin DMA, Clamp M, Barton GJ. 2009. Jalview Version 2—a multiple sequence alignment editor and analysis workbench. Bioinformatics 25:1189–1191. 10.1093/bioinformatics/btp033. 19151095 PMC2672624

[56] Price MN, Dehal PS, Arkin AP. 2009. FastTree: computing large minimum evolution trees with profiles instead of a distance matrix. Mol Biol Evol 26:1641–1650. 10.1093/molbev/msp077. 19377059 PMC2693737

[57] Stamatakis A. 2014. RAxML version 8: a tool for phylogenetic analysis and post-analysis of large phylogenies. Bioinformatics 30:1312–1313. 10.1093/bioinformatics/btu033. 24451623 PMC3998144

[58] Asnicar F, Weingart G, Tickle TL, Huttenhower C, Segata N. 2015. Compact graphical representation of phylogenetic data and metadata with GraPhlAn. PeerJ 3:e1029. 10.7717/peerj.1029. 26157614 PMC4476132

[59] Asnicar F, Thomas AM, Beghini F, Mengoni C, Manara S, Manghi P, Zhu Q, Bolzan M, Cumbo F, May U, Sanders JG, Zolfo M, Kopylova E, Pasolli E, Knight R, Mirarab S, Huttenhower C, Segata N. 2020. Precise phylogenetic analysis of microbial isolates and genomes from metagenomes using PhyloPhlAn 3.0. Nat Commun 11:2500. 10.1038/s41467-020-16366-7. 32427907 PMC7237447

[60] Sand A, Holt MK, Johansen J, Brodal GS, Mailund T, Pedersen CNS. 2014. tqDist: a library for computing the quartet and triplet distances between binary or general trees. Bioinformatics 30:2079–2080. 10.1093/bioinformatics/btu157. 24651968

[61] Langmead B, Salzberg SL. 2012. Fast gapped-read alignment with Bowtie 2. Nat Methods 9:357–359. 10.1038/nmeth.1923. 22388286 PMC3322381

[62] Li H, Handsaker B, Wysoker A, Fennell T, Ruan J, Homer N, Marth G, Abecasis G, Durbin R, 1000 Genome Project Data Processing Subgroup. 2009. The sequence alignment/map format and SAMtools. Bioinformatics 25:2078–2079. 10.1093/bioinformatics/btp352. 19505943 PMC2723002

[63] Danecek P, Auton A, Abecasis G, Albers CA, Banks E, DePristo MA, Handsaker RE, Lunter G, Marth GT, Sherry ST, McVean G, Durbin R, 1000 Genomes Project Analysis Group. 2011. The variant call format and VCFtools. Bioinformatics 27:2156–2158. 10.1093/bioinformatics/btr330. 21653522 PMC3137218

[64] Truong DT, Franzosa EA, Tickle TL, Scholz M, Weingart G, Pasolli E, Tett A, Huttenhower C, Segata N. 2015. MetaPhlAn2 for enhanced metagenomic taxonomic profiling. Nat Methods 12:902–903. 10.1038/nmeth.3589. 26418763

[65] Segata N, Waldron L, Ballarini A, Narasimhan V, Jousson O, Huttenhower C. 2012. Metagenomic microbial community profiling using unique clade-specific marker genes. Nat Methods 9:811–814. 10.1038/nmeth.2066. 22688413 PMC3443552

[66] Costea PI, Zeller G, Sunagawa S, Pelletier E, Alberti A, Levenez F, Tramontano M, Driessen M, Hercog R, Jung F-E, Kultima JR, Hayward MR, Coelho LP, Allen-Vercoe E, Bertrand L, Blaut M, Brown JRM, Carton T, Cools-Portier S, Daigneault M, Derrien M, Druesne A, de Vos WM, Finlay BB, Flint HJ, Guarner F, Hattori M, Heilig H, Luna RA, van Hylckama Vlieg J, Junick J, Klymiuk I, Langella P, Le Chatelier E, Mai V, Manichanh C, Martin JC, Mery C, Morita H, O'Toole PW, Orvain C, Patil KR, Penders J, Persson S, Pons N, Popova M, Salonen A, Saulnier D, Scott KP, Singh B, et al. 2017. Towards standards for human fecal sample processing in metagenomic studies. Nat Biotechnol 35:1069–1076. 10.1038/nbt.3960. 28967887

[67] Dhakan DB, Maji A, Sharma AK, Saxena R, Pulikkan J, Grace T, Gomez A, Scaria J, Amato KR, Sharma VK. 2019. The unique composition of Indian gut microbiome, gene catalogue, and associated fecal metabolome deciphered using multi-omics approaches. Gigascience 8. 10.1093/gigascience/giz004. PMC6394208 30698687

[68] Hansen LBS, Roager HM, Søndertoft NB, Gøbel RJ, Kristensen M, Vallès-Colomer M, Vieira-Silva S, Ibrügger S, Lind MV, Mærkedahl RB, Bahl MI, Madsen ML, Havelund J, Falony G, Tetens I, Nielsen T, Allin KH, Frandsen HL, Hartmann B, Holst JJ, Sparholt MH, Holck J, Blennow A, Moll JM, Meyer AS, Hoppe C, Poulsen JH, Carvalho V, Sagnelli D, Dalgaard MD, Christensen AF, Lydolph MC, Ross AB, Villas-Bôas S, Brix S, Sicheritz-Pontén T, Buschard K, Linneberg A, Rumessen JJ, Ekstrøm CT, Ritz C, Kristiansen K, Nielsen HB, Vestergaard H, Færgeman NJ, Raes J, Frøkiær H, Hansen T, Lauritzen L, Gupta R, Licht TR, et al. 2018. A low-gluten diet induces changes in the intestinal microbiome of healthy Danish adults. Nat Commun 9:4630. 10.1038/s41467-018-07019-x. 30425247 PMC6234216

[69] Schirmer M, Smeekens SP, Vlamakis H, Jaeger M, Oosting M, Franzosa EA, Horst RT, Jansen T, Jacobs L, Bonder MJ, Kurilshikov A, Fu J, Joosten LAB, Zhernakova A, Huttenhower C, Wijmenga C, Netea MG, Xavier RJ. 2016. Linking the human gut microbiome to inflammatory cytokine production capacity. Cell 167:1897. 10.1016/j.cell.2016.11.046. 27984736

[70] Zeller G, Tap J, Voigt AY, Sunagawa S, Kultima JR, Costea PI, Amiot A, Böhm J, Brunetti F, Habermann N, Hercog R, Koch M, Luciani A, Mende DR, Schneider MA, Schrotz-King P, Tournigand C, Tran Van Nhieu J, Yamada T, Zimmermann J, Benes V, Kloor M, Ulrich CM, von Knebel Doeberitz M, Sobhani I, Bork P. 2014. Potential of fecal microbiota for early-stage detection of colorectal cancer. Mol Syst Biol 10:766. 10.15252/msb.20145645. 25432777 PMC4299606

