# Supplementary material for: Genes Encoding Microbial Acyl Coenzyme A Binding Protein/Diazepam-Binding Inhibitor Orthologs Are Rare in the Human Gut Microbiome and Show No Links to Obesity
Source: Appl Environ Microbiol. 2021 May 26;87(12):e00471-21. doi: 10.1128/AEM.00471-21 (PMC8174751; doi:10.1128/AEM.00471-21)
Supplement: SUPPLEMENTAL FILE 1 — Supplemental material. Download aem.00471-21-s0001.pdf, PDF file, 1.1 MB [file aem.00471-21-s0001.pdf]

Supplementary Figures

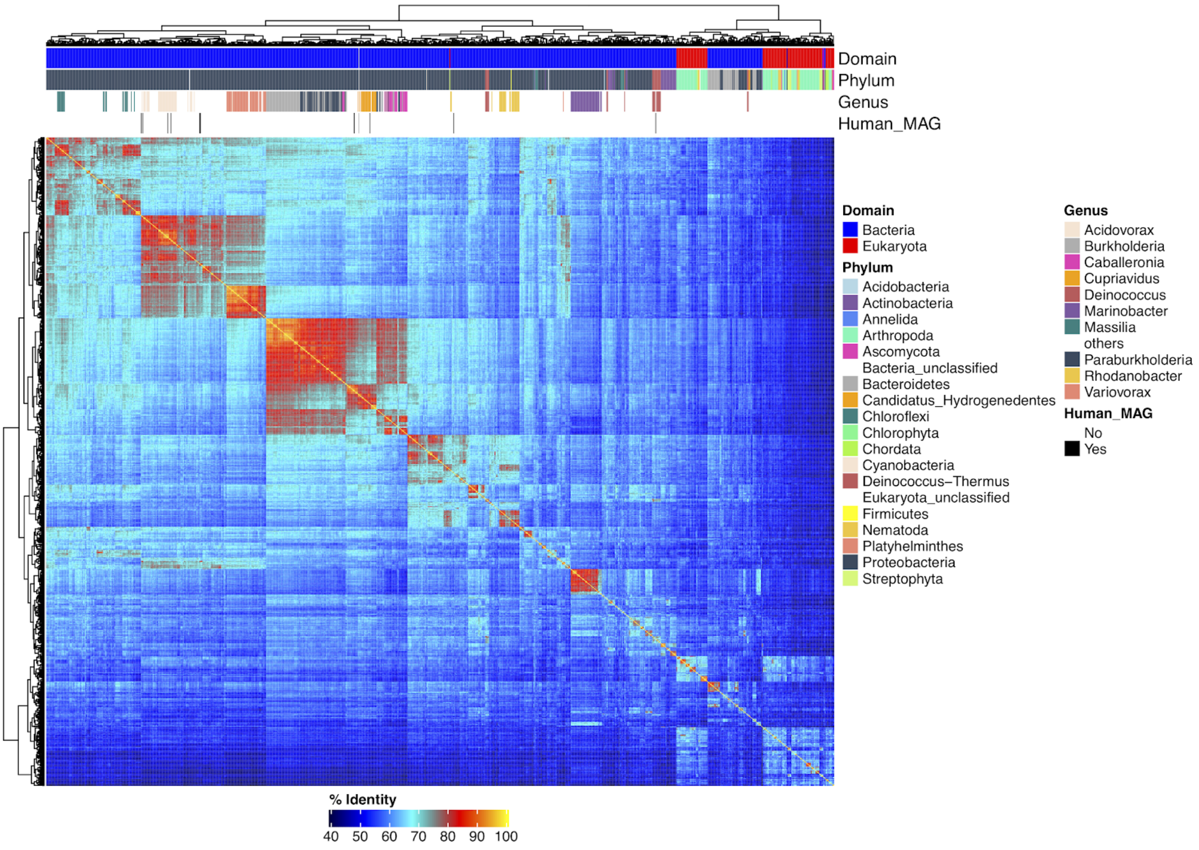

Supplementary Figure 1. Pairwise nucleotide identity of retrieved ACBP/DBI sequences.

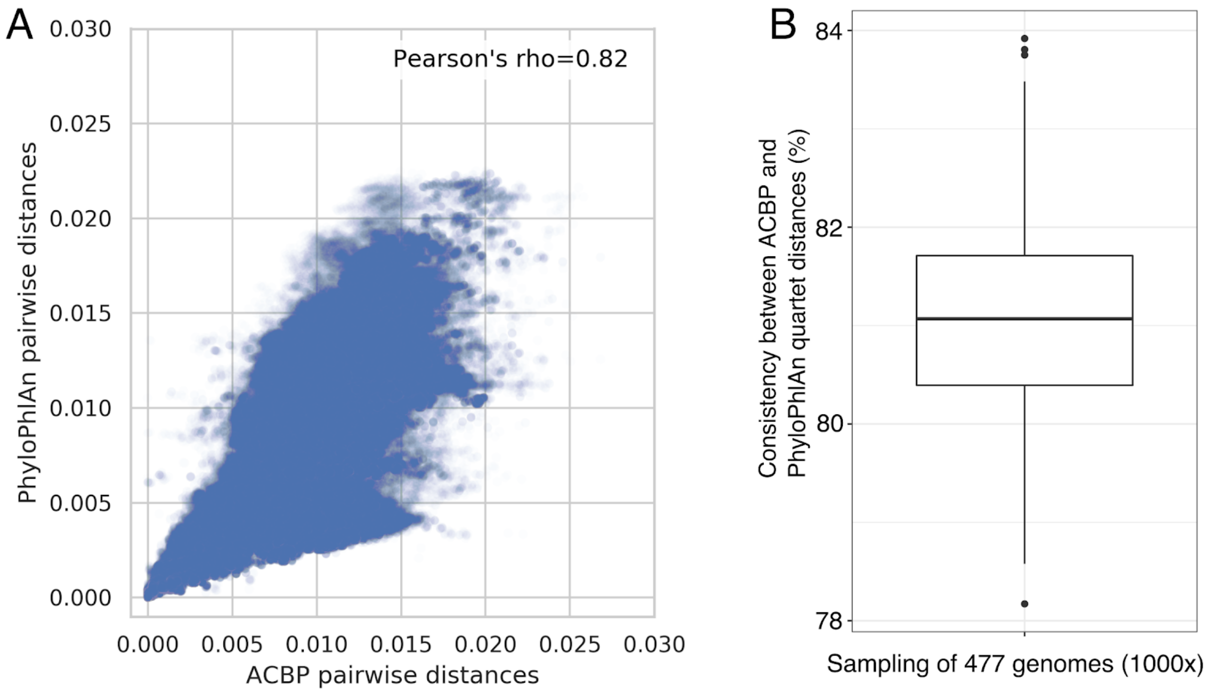

Supplementary Figure 2. Comparison of phylogenetic trees built using either PhyloPhlAn 3 (with PhyloPhlAn markers) or retrieved ACBP/DBI sequences on a set of 3,490 reference prokaryotic genomes and 129 MAGs.
